# Insights from long-term follow-up of patients treated with oral immunotherapy for cow's milk allergy

**DOI:** 10.31744/einstein_journal/2026AO2168

**Published:** 2026-08-12

**Authors:** Lais Ferreira Lopes Brum, Nayara Maria Furquim Nasser, Glauce Hiromi Yonamine, Thais Costa Lima de Moura, Mayra de Barros Dorna, Antonio Carlos Pastorino, Beni Morgenstern, Ana Paula Moschione Castro

**Affiliations:** 1 Universidade de São Paulo Hospital das Clínicas Faculdade de Medicina Unidade de Alergia e Imunologia do Instituto da Criança e do Adolescente São Paulo SP Brazil Unidade de Alergia e Imunologia do Instituto da Criança e do Adolescente, Hospital das Clínicas Faculdade de Medicina, Universidade de São Paulo, São Paulo, SP, Brazil.

**Keywords:** Food hypersensitivity, Desensitization, immunologic, Administration, oral, Immunotherapy, Allergen immunotherapy, Milk hypersensitivity, Oral immunotherapy, Hypersensitivity, immediate

## Abstract

**Objective:**

To describe the long-term outcomes of patients undergoing oral immunotherapy for cow's milk allergy in a pediatric allergy clinic, focusing on treatment efficacy and adverse reactions over 5 years of follow-up.

**Methods:**

This retrospective cohort study reviewed medical records of patients aged 5-18 years who underwent cow's milk oral immunotherapy at *Instituto da Criança*, São Paulo. All patients initiated treatment between 2013 and 2019 and completed at least 5 years of maintenance follow-up. Evaluated outcomes included treatment adherence, adverse reactions, anaphylaxis, and eosinophilic esophagitis. Data were analyzed descriptively, and statistical tests were applied.

**Results:**

Thirty patients entered the maintenance phase (median age, 8.2 years). Most had allergic comorbidities and a history of anaphylaxis. Four patients discontinued treatment within the first trimester: two due to anaphylaxis and two due to eosinophilic esophagitis. Adverse reactions were predominantly mild and decreased significantly over time (p=0.01). Severe reactions accounted for 17% of events, including 22 episodes of anaphylaxis. Eosinophilic esophagitis was diagnosed in 33% of patients and occurred more frequently among those initiating treatment after 12 years of age.

**Conclusion:**

Long-term oral immunotherapy increased tolerance and enabled regular milk ingestion in most patients. Older age at treatment initiation was associated with a higher incidence of eosinophilic esophagitis. Careful patient selection and structured monitoring during and after treatment are essential for early identification of severe reactions and eosinophilic esophagitis. Larger studies with longer follow-up are needed to optimize protocols and improve safety strategies.

## Highlights

Cow's milk oral immunotherapy maintained desensitization over five years.

Anaphylactic reactions were more frequent during the first maintenance year.

The risk of eosinophilic esophagitis persisted throughout follow-up.

Continuous monitoring for allergic reactions and esophagitis symptoms is required.

## In Brief

The study described long-term outcomes of oral immunotherapy for cow's milk allergy. Over 5 years, 33% of patients developed eosinophilic esophagitis. Anaphylaxis and mild reactions were more frequent during the first maintenance year and decreased over time, reinforcing the need for continuous monitoring.

## Graphical abstract

**Figure:**
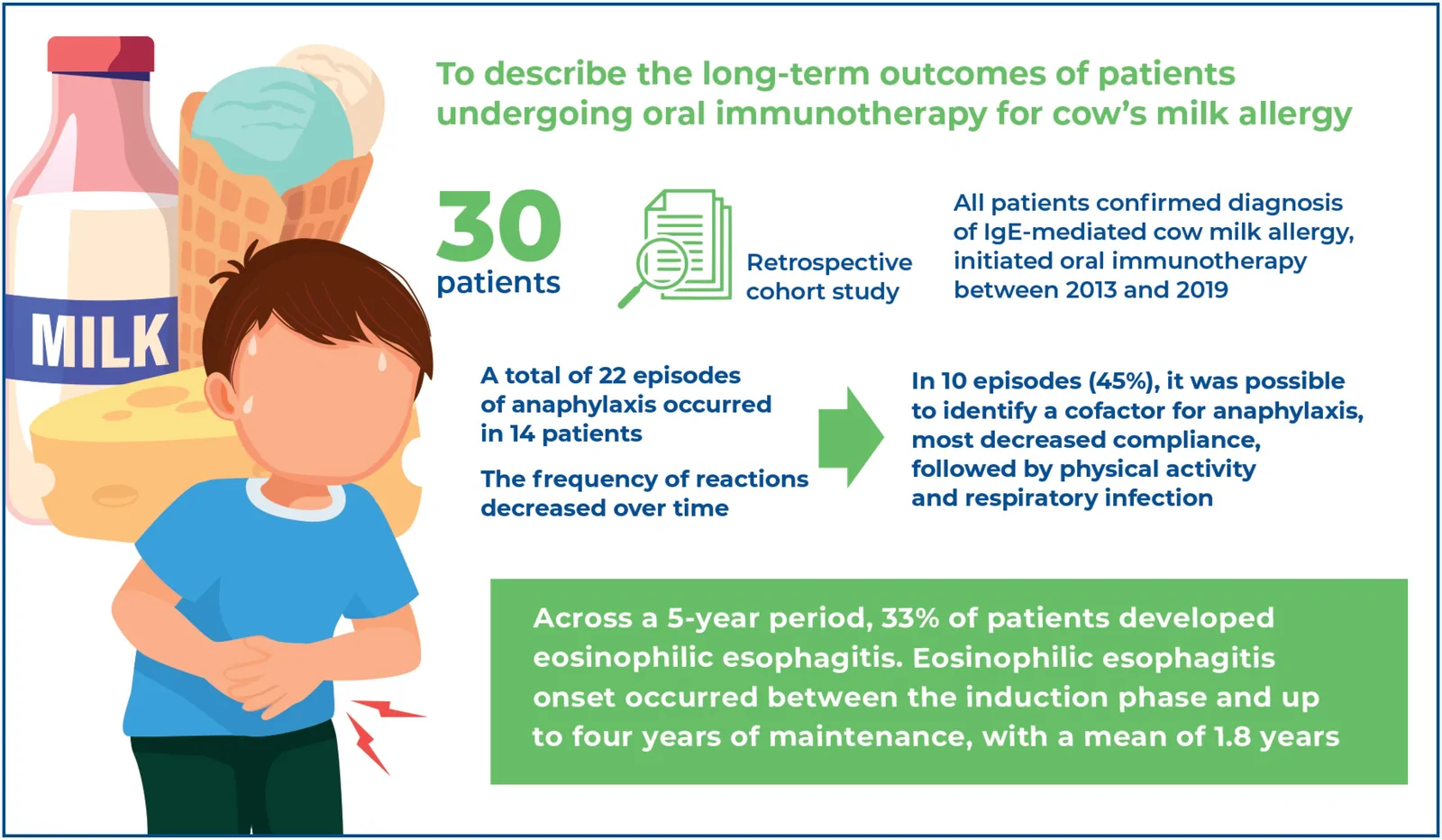

## INTRODUCTION

Immunoglobulin E (IgE)-mediated cow's milk allergy (CMA) is the most common food allergy in infants, affecting approximately 2% of children under 4 years of age worldwide.^(1)^ However, prevalence rates confirmed by oral food challenges range from 0.21% to 4.9%.^(2-6)^

Although the prognosis is generally favorable, with most children eventually acquiring tolerance, a subset of patients may experience persistent CMA into adulthood.^(7,8)^ In this context, oral immunotherapy (OIT) has emerged as a potential strategy to enable the ingestion of allergenic foods. A systematic review conducted for the World Allergy Organization DRACMA (Diagnosis and Rationale for Action against Cow's Milk Allergy) guideline reported a higher likelihood of desensitization (DS), *i.e.*, safe milk consumption following OIT, compared with an elimination diet.^(9)^ Accordingly, allergen-specific OIT has been proposed as a promising therapeutic option and has already been implemented in several reference centers.^(10,11)^ Oral immunotherapy for CMA consists of the titrated oral administration of cow's milk at regular intervals to modulate the specific immune response to milk proteins in patients with CMA.

The outcomes of OIT are usually measured as DS and sustained unresponsiveness (SU).^(12)^ Desensitization refers to an increased reaction threshold, *i.e.*, the amount of allergen that can be ingested without triggering a reaction. This temporary state is maintained through continuous, usually daily, allergen exposure.^(13)^ Sustained unresponsiveness is defined as the ability to tolerate the allergen after a defined period of avoidance (typically 4 to 8 weeks) following discontinuation of OIT, without clinical symptoms.^(12)^ In contrast, remission refers to a more durable absence of clinical reactivity after discontinuation of allergen ingestion, suggesting a more persistent immunologic change. However, few studies have described the long-term outcomes of patients undergoing OIT, particularly for CMA.

## OBJECTIVE

The objective of this study was to describe the long-term outcomes of patients undergoing oral immunotherapy for cow's milk in a pediatric allergy clinic, with emphasis on treatment efficacy and adverse reactions after 5 years of follow-up.

## METHODS

This retrospective observational cohort study was based on the review of medical records of patients followed at the Food Allergy Outpatient Clinic of the Allergy and Immunology Unit, *Instituto da Criança, Hospital das Clínicas, São Paulo*. The study evaluated the efficacy and safety of maintenance OIT for cow's milk.

Inclusion criteria comprised patients aged 5 to 18 years with a confirmed diagnosis of IgE-mediated CMA, defined by either a positive oral food challenge or a documented clinical reaction within the year preceding OIT initiation. All patients underwent cow's milk oral immunotherapy (CM-OIT) between 2013 and 2019 and therefore had at least 5 years of maintenance follow-up. Patients who were unable to complete the build-up phase and reach maintenance due to any intercurrence were excluded.

All participants underwent the same standardized CM-OIT protocol, based exclusively on fresh milk. No patients received baked milk OIT as a separate modality, and no mixed or step-up approaches transitioning from baked milk to fresh milk were used; therefore, treatment outcomes reflected responses to a uniform protocol. The CM-OIT protocol consisted of an induction phase with daily milk ingestion and gradual weekly dose escalation. The induction phase began with an extremely diluted cow's milk solution (1:10^6^), followed by progressive weekly increases in concentration until full-strength milk was achieved. Incremental volume increases were then introduced, progressing toward a target dose of 200mL of fresh whole cow's milk per day, which defined entry into the maintenance phase.

During maintenance, patients were instructed to ingest 180-200mL of milk daily, while consumption of other milk-containing foods was permitted. After a minimum of 3 years of maintenance, SU was assessed by complete withdrawal of cow's milk for 4 weeks, followed by a supervised oral food challenge with 200 mL of fresh whole cow's milk.

Evaluated outcomes included treatment adherence, defined as intake of more than 70% of the prescribed daily dose of 200mL of fresh cow's milk during the evaluation period; adverse reactions reported by patients; anaphylaxis, defined as the sudden onset of symptoms involving at least two organ systems;^(14)^ and eosinophilic esophagitis (EoE), defined by compatible clinical symptoms associated with endoscopic findings of >15 eosinophils/high-power field.^(15)^ Mild allergic reactions were defined according to Sampson et al. as symptoms limited to a single organ system, including localized cutaneous manifestations (urticaria, erythema, pruritus), mild upper respiratory symptoms (rhinorrhea, sneezing, cough), oral pruritus, or mild gastrointestinal symptoms (nausea or abdominal discomfort). Results were presented descriptively over the 5-year follow-up period. Statistical analyses were performed using the Cochran-Armitage trend test, the χ^2^ test, and Fisher's exact test for categorical data.

## RESULTS

Thirty patients (14 M:16 F) entered the maintenance phase between 5.0 and 15.9 years of age (median, 8.2 years). All had confirmed IgE-mediated CMA and a documented history of anaphylaxis. Twenty-six patients (73.3%) presented with at least one allergic comorbidity (Table 1). Four patients discontinued CM-OIT within the first trimester of follow-up: two due to anaphylactic reactions and two due to EoE confirmed by endoscopy. Five additional patients discontinued follow-up over time. Overall, 21 patients (70%) maintained regular cow's milk ingestion.

**Table 1:** Characteristics of patients with cow's milk allergy

Characteristics		
Age at OIT initiation, years, median (range)		8.25 (5-15,9)
Sex - n (%)		
	Male	14 (46.6)
	Female	16 (53.4)
Allergic comorbidities - n (%)		
	Asthma	17 (56.6)
	Allergic rhinitis	22 (73.3)
	Atopic dermatitis	6 (26)
	Other food allergy	8 (26.6)
History of anaphylaxis to cow's milk before OIT, n (%)		30 (100)
Specific IgE (kU/L), median (range) before OIT		
	Cow's milk	38.6 (0.52-100)
	Casein	46.7 (0.7-100)
	β-lactoglobulin	4.25 (0.1-100)
	α-lactalbumin	7.25 (0.1-100)

CMA: cow's milk allergy; OIT: oral immunotherapy; IgE: immunoglobulin E.

Patients who remained in the maintenance phase ingested 180-200mL of fresh whole cow's milk daily, either as a single dose or divided into two 100mL portions, in addition to consuming milk-containing foods. SU was assessed in six patients after a mean maintenance period of 5.36 years (minimum, 3 years and 7 months). Five patients achieved SU and discontinued the daily 200mL milk intake while maintaining only unrestricted consumption of milk-containing foods without allergic reactions following the oral food challenge. The patient who did not achieve SU was 13 years and 9 months old at the time of testing and had completed 5.5 years of maintenance. This patient experienced mild symptoms (urticaria and oropharyngeal pruritus) after ingestion of 60mL of milk, was retreated according to the CM-OIT protocol, and resumed daily consumption of 200mL of milk without further complications.

Reactions related to OIT were classified as immediate, with symptoms occurring within 2 hours after ingestion, or delayed, predominantly gastrointestinal symptoms without a direct temporal association with milk intake but potentially related to CM-OIT, including nausea, abdominal pain, retrosternal pain, and food impaction.

Immediate reactions were classified as mild (82%) or severe (17.4%). Mild reactions involved a single organ system, predominantly the skin (70.8%), followed by the gastrointestinal tract (16.5%) and respiratory symptoms. Cutaneous manifestations included urticaria, rash, and isolated pruritus; gastrointestinal symptoms included nausea and abdominal pain; and cough was the most frequent respiratory manifestation.

A total of 22 episodes of anaphylaxis occurred in 14 patients. In 10 episodes (45%), a cofactor was identified, most commonly decreased compliance (missed doses or milk ingestion after fasting), followed by physical activity and respiratory infection. Only five patients (16%) did not experience adverse reactions during the 5-year follow-up period.

The frequency of reactions decreased over time. The first year showed the highest number of mild reactions, with a mean of 1.8 reactions per patient, whereas only two patients experienced mild reactions after 5 years of follow-up. Anaphylactic reactions occurred significantly more frequently during the first year than during the fifth year (p=0.01, Cochran-Armitage trend test).

During follow-up, 10 patients (5 M, 5 F) developed EoE symptoms confirmed by endoscopy, corresponding to 33% of the cohort. Age at diagnosis ranged from 6.9 to 17.7 years (median, 14.8). Eosinophilic esophagitis onset occurred between the induction phase and the fourth year of maintenance, with a mean onset time of 1.8 years. In five patients, EoE developed during the first year of maintenance, three of whom remained on OIT. In two patients, EoE developed during the fifth year of follow-up. Older patients (>12 years) were more likely to develop EoE (p<0.05), whereas the occurrence of anaphylactic reactions during maintenance was not associated with EoE frequency (χ^2^ test) (Table 2 or Figure 1).

**Table 2:** Distribution of mild reactions, anaphylaxis, EoE, and follow-up status during CM-OIT maintenance

Maintenance duration	1 Year	2 Years	3 Years	4 Years	5 Years
Mild reactions (episodes)	49	33	10	7	5
Mild reactions (patients)	15	13	6	4	2
Anaphylaxis (episodes)	12	3	3	3	1
Anaphylaxis (patients)	10	3	3	2	1
EoE (diagnosis)	5	0	2	1	2
Discontinuation	4	0	0	0	0
Lost to follow-up	2	1	1	1	0
Total patients	30	24	23	22	21

EoE, eosinophilic esophagitis.

**Figure 1:**
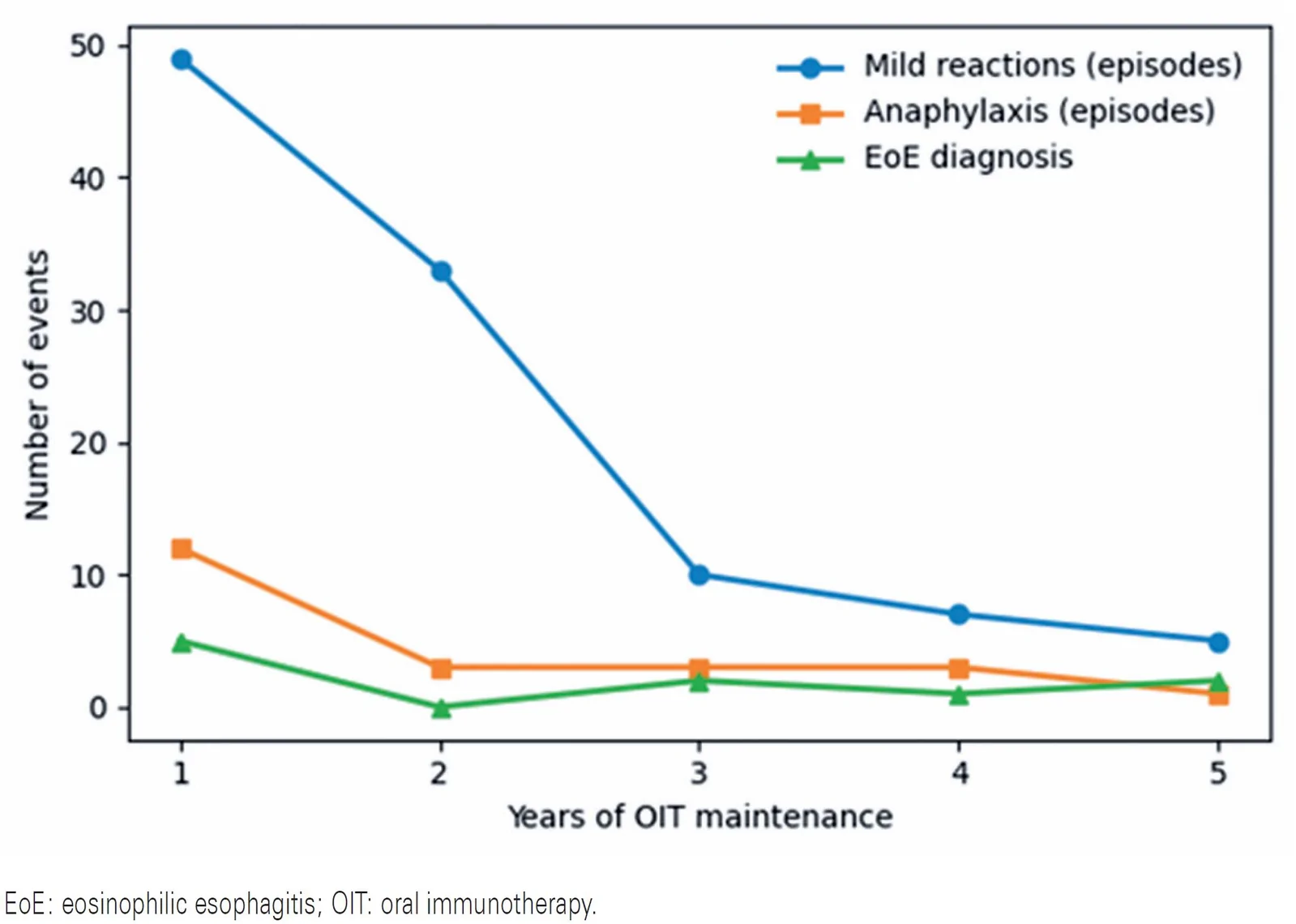
Annual progression of mild reactions, episodes of anaphylaxis, and diagnoses of eosinophilic esophagitis during 5 years of oral immunotherapy maintenance

EoE: eosinophilic esophagitis; OIT: oral immunotherapy.

## DISCUSSION

This study reports the long-term follow-up of patients who underwent CM-OIT, highlighting outcomes in this age group, as CM-OIT is becoming increasingly common among allergic patients. Oral immunotherapy was primarily indicated for patients with IgE-mediated CMA at higher risk of severe reactions and with a history of anaphylaxis. The analysis focused on the maintenance phase, during which daily ingestion of a predetermined amount of milk, approximately 200mL of fresh milk, is recommended. Most patients adhered to the regimen and continued regular milk consumption.

Most patients undergoing CM-OIT achieve DS regardless of the protocol used. However, there is heterogeneity in outcomes and adverse reactions, and new treatment strategies have emerged in recent years, with recent studies emphasizing early initiation of CM-OIT.^(16)^ In our cohort, CM-OIT effectively maintained DS over 5 years in most patients. The discontinuation rate was low (13%), with the first year appearing critical, as adverse events, including anaphylaxis and EoE, were the main reasons for early withdrawal.

A retrospective study comparing high- (200mL) and low-dose (3mL) maintenance protocols in children with anaphylactic CMA reported discontinuation rates after 3 years of 24% and 11%, respectively, suggesting that lower-dose protocols may improve long-term adherence.^(17)^ Systematic reviews have also reported discontinuation rates ranging from 9% to <20%, generally related to severe or recurrent adverse events during maintenance.^(18)^

Initiating CM-OIT in school-age children and adolescents, as observed in our study, is currently considered late according to more recent evidence. Recent studies have shown favorable outcomes with early-start OIT, even before 4 years of age. However, these findings may be influenced by the possibility of natural tolerance development. Nevertheless, two studies including control groups subjected to strict milk exclusion demonstrated significantly higher tolerance following early introduction compared with controls.^(19,20)^

Accordingly, there is a growing trend toward earlier OIT initiation because of increased efficacy and the potential for fewer adverse events. In our study, patients initiating OIT after 12 years of age showed a higher incidence of EoE. Data regarding the specific risk of EoE with very early CM-OIT initiation (<2 years of age) remain limited, although EoE has been reported in infants and young children, including those with a history of cow's milk protein allergy. In children younger than 2 years, EoE symptoms may be nonspecific, including feeding difficulties, vomiting, cough, and respiratory symptoms, which may delay diagnosis.^(21)^

The main concern during follow-up is the occurrence of adverse events, particularly because these patients often have severe allergic disease, as observed in our cohort, in which all patients had a history of anaphylaxis and a high prevalence of other allergic diseases, especially atopic dermatitis. Few studies have specifically evaluated the maintenance phase of CM-OIT, and findings regarding adverse events remain inconsistent. In the study by Ines Mota and colleagues, 45% of patients developed mild-to-severe allergic reactions during maintenance, although only 7% experienced more than three episodes; however, only 36% of the cohort had a history of anaphylaxis.^(22)^ Therefore, clinicians should be prepared for IgE-mediated reactions ranging from mild to severe, and carrying an epinephrine auto-injector throughout treatment is strongly recommended. It is also important to recognize that adverse reactions may occur even in the absence of OIT. One study following patients with CMA until tolerance development or 18 years of age found that at least one-third experienced allergic reactions due to accidental ingestion of cow's milk-containing foods.^(16)^

EoE has been reported as a late adverse effect in OIT.^(23)^ The reported prevalence of EoE during CM-OIT varies across studies. The DRACMA guidelines describe a biopsy-confirmed EoE rate of 5% among patients undergoing treatment.^(24)^ In our study, the prevalence reached 33%, exceeding rates previously reported in the literature. In Morales-Cabeza's study, EoE developed after a mean of 2.8 years of maintenance,^(25)^ whereas in our cohort the mean time to diagnosis was 1.8 years. However, one patient was diagnosed during the fourth year of maintenance, reinforcing the importance of long-term follow-up.

After 3 years of maintenance, patients were offered a 4-week elimination period to assess SU. In our study, only five patients agreed to testing, and SU was demonstrated in four. Temporary elimination of cow's milk has been proposed as a strategy to distinguish DS from SU.^(26)^ However, in real-world practice, this approach is not fully accepted by patients and caregivers. While some prefer to maintain daily milk consumption, others experience fear and anxiety regarding symptom recurrence after reintroduction. In Brazil, the widespread use of milk in homemade foods and desserts may also contribute to reluctance toward elimination testing.

Long-term follow-up of patients undergoing OIT is essential to assess efficacy, safety, and treatment adherence. Longitudinal studies have shown that even after completing OIT, many patients continue to experience adverse reactions, although these generally decrease in severity and frequency over time, and some cases may still require epinephrine for severe reactions.^(27)^ The progressive reduction in adverse reactions and immunologic reactivity, demonstrated by laboratory markers, further supports prolonged monitoring to evaluate DS and identify patients at risk of losing tolerance.^(28)^

A major strength of this study was the prolonged follow-up, which demonstrated that the risk of anaphylaxis is substantially higher during the first year of maintenance, whereas the risk of EoE persists throughout follow-up, even after more than 3 years of maintenance. Therefore, EoE symptoms should be assessed at every follow-up visit, and upper endoscopy should be performed when clinically indicated.

This descriptive study has several limitations, including the lack of protocol uniformity across studies. Although a build-up phase followed by maintenance is widely recommended, escalation rates, administration methods, and maintenance doses vary considerably, limiting direct comparisons. In addition, analyses correlating age at treatment initiation with mild adverse reactions were limited by sample size, non-uniform follow-up, and the exploratory nature of the analyses. The absence of clinically meaningful associations suggests that mild reaction frequency was not primarily driven by age or exposure duration, reinforcing the descriptive safety focus of the cohort. Additional limitations include the absence of a control group and variability in outcomes, as patients and families were encouraged to make autonomous decisions, including discontinuing follow-up. Nevertheless, this study contributes to a clinically relevant field with still limited long-term data.

## CONCLUSION

Cow's milk-oral immunotherapy is effective in desensitizing patients to cow's milk proteins; however, the heterogeneity of outcomes and occurrence of adverse reactions underscore the need for individualized strategies. Later treatment initiation was associated with a higher incidence of adverse events, particularly eosinophilic esophagitis. Therefore, the decision to initiate cow's milk-oral immunotherapy should consider not only patient age but also clinical profile and adherence potential. Rigorous follow-up is recommended during and after treatment, with emphasis on early detection of severe reactions and assessment of sustained unresponsiveness. Larger studies with longer follow-up are needed to optimize protocols for different patient profiles, clarify the mechanisms underlying tolerance maintenance, and determine the duration of protection in older patients.

## Data Availability

The underlying content is contained within the manuscript.

## References

[1] Rona RJ, Keil T, Summers C, Gislason D, Zuidmeer L, Sodergren E (2007). The prevalence of food allergy: a meta-analysis. J Allergy Clin Immunol.

[2] Høst A, Halken S, Jacobsen HP, Christensen AE, Herskind AM, Plesner K (2002). Clinical course of cow's milk protein allergy/intolerance and atopic diseases in childhood. Pediatr Allergy Immunol.

[3] Saarinen KM, Juntunen-Backman K, Järvenpää AL, Kuitunen P, Lope L, Renlund M (1999). Supplementary feeding in maternity hospitals and the risk of cow's milk allergy: A prospective study of 6209 infants. J Allergy Clin Immunol.

[4] Kvenshagen B, Halvorsen R, Jacobsen M (2008). Adverse reactions to milk in infants. Acta Paediatr.

[5] Venter C, Pereira B, Grundy J, Clayton CB, Arshad SH, Dean T (2006). Prevalence of sensitization reported and objectively assessed food hypersensitivity amongst six-year-old children: a population-based study. Pediatr Allergy Immunol.

[6] Schrander JJ, van den Bogart JP, Forget PP, Schrander-Stumpel CT, Kuijten RH, Kester AD (1993). Cow's milk protein intolerance in infants under 1 year of age: a prospective epidemiological study. Eur J Pediatr.

[7] Santos A, Dias A, Pinheiro JA (2010). Predictive factors for the persistence of cow's milk allergy. Pediatr Allergy Immunol.

[8] Skripak JM, Matsui EC, Mudd K, Wood RA (2007). The natural history of IgE-mediated cow's milk allergy. J Allergy Clin Immunol.

[9] Brożek JL, Terracciano L, Hsu J, Kreis J, Compalati E, Santesso N (2012). Oral immunotherapy for IgE-mediated cow's milk allergy: a systematic review and meta-analysis. Clin Exp Allergy.

[10] Skripak JM, Nash SD, Rowley H, Brereton NH, Oh S, Hamilton RG (2008). A randomized, double-blind, placebo-controlled study of milk oral immunotherapy for cow's milk allergy. J Allergy Clin Immunol.

[11] Zapatero L, Alonso E, Fuentes V, Martínez MI (2008). Oral desensitization in children with cow's milk allergy. J Investig Allergol Clin Immunol.

[12] Sampson HA, O’Mahony L, Burks AW, Plaut M, Lack G, Akdis CA (2018). Mechanisms of food allergy. J Allergy Clin Immunol.

[13] Chinthrajah RS, Purington N, Andorf S, Long A, O’Laughlin KL, Lyu SC (2019). Sustained outcomes in oral immunotherapy for peanut allergy (POISED study): a large, randomised, double-blind, placebo-controlled, phase 2 study. Lancet.

[14] Cardona V, Ansotegui IJ, Ebisawa M, El-Gamal Y, Fernandez Rivas M, Fineman S (2020). World allergy organization anaphylaxis guidance 2020. World Allergy Organ J.

[15] Dellon ES, Liacouras CA, Molina-Infante J, Furuta GT, Spergel JM, Zevit N (2018). Updated International Consensus Diagnostic Criteria for Eosinophilic Esophagitis: proceedings of the AGREE Conference. Gastroenterology.

[16] Lebrero EA, Moya LF, Somoza Alvarez ML (2001). Alergia a leche y huevo en ninos. Alergol Immunol Clin.

[17] Ito Y, Nagakura KI, Sato S, Ebisawa M, Yanagida N (2025). Long-term comparison of high- and low-dose oral immunotherapy in children with anaphylactic cow's milk allergy. Pediatr Allergy Immunol.

[18] Pajno GB, Caminiti L, Chiera F, Crisafulli G, Salzano G, Arasi S (2016). Safety profile of oral immunotherapy with cow's milk and hen egg: A 10-year experience in controlled trials. Allergy Asthma Proc.

[19] Nowak-Wegrzyn A, Sato S, Fiocchi A, Ebisawa M (2019). Oral and sublingual immunotherapy for food allergy. Curr Opin Allergy Clin Immunol.

[20] Vickery BP, Berglund JP, Burk CM, Fine JP, Kim EH, Kim JI (2017). Early oral immunotherapy in infants with milk allergy: a pilot study. J Allergy Clin Immunol.

[21] Wilson BE, Sacta MA, Wright BL, Spergel J, Wolfset N (2024). The relationship between eosinophilic esophagitis and immunotherapy. Immunol Allergy Clin North Am.

[22] Mota I, Piedade S, Gaspar Â, Benito-Garcia F, Sampaio G, Borrego LM (2018). Cow's milk oral immunotherapy in real life: 8-year long-term follow-up study. Asia Pac Allergy.

[23] Wang Y, Liu S, Lu M, Guo J, Lv C, Huang L (2025). Oral immunotherapy for cow's milk allergy in children: a systematic review and meta-analysis. Front Immunol.

[24] Fiocchi A, Pawankar R, Cuello-Garcia C, Ahn K, Al-Hammadi S, Agarwal A (2023). World Allergy Organization (WAO) Diagnosis and Rationale for Action against Cow's Milk Allergy (DRACMA) Guidelines update - VII - Milk elimination and reintroduction in the diagnostic process of cow's milk allergy. World Allergy Organ J.

[25] Morales-Cabeza C, Infante S, Cabrera-Freitag P, Fuentes-Aparicio V, Zubeldia JM, Álvarez-Perea A (2023). Oral immunotherapy and risk of eosinophilic esophagitis in children: 15 years’ experience. J Pediatr Gastroenterol Nutr.

[26] Badina L, Levantino L, Carrato V, Peruch G, Celsi F, Barbi E (2021). Early introduction oral immunotherapy for IgE-mediated cow's milk allergy: A follow-up study confirms this approach as safe and appealing to parents. Immun Inflamm Dis.

[27] Nachshon L, Goldberg MR, Katz Y, Levy MB, Elizur A (2018). Long-term outcome of peanut oral immunotherapy-Real-life experience. Pediatr Allergy Immunol.

[28] Elizur A, Appel MY, Goldberg MR, Yichie T, Levy MB, Nachshon L (2016). Clinical and laboratory 2-year outcome of oral immunotherapy in patients with cow's milk allergy. Allergy.

